# Estimation of moral distress among nurses: A systematic review and meta-analysis

**DOI:** 10.1177/09697330221135212

**Published:** 2023-01-27

**Authors:** Zainab Alimoradi, Elahe Jafari, Chung-Ying Lin, Raheleh Rajabi, Zohreh Hosseini Marznaki, Mostafa Soodmand, Marc N Potenza, Amir H Pakpour

**Affiliations:** 113106 Qazvin University of Medical Sciences, Qazvin, Iran; Institute of Allied Health Sciences, College of Medicine, 34912 National Cheng Kung University, Taiwan; Biostatistics Consulting Center, National Cheng Kung University Hospital, College of Medicine, National Cheng Kung University, Taiwan; Department of Occupational Therapy, College of Medicine, National Cheng Kung University, Taiwan; Department of Public Health, College of Medicine, National Cheng Kung University, Taiwan; 48463 Kerman University of Medical Sciences, Iran; 108890 Mazandaran University of Medical Sciences, Iran; Guilan University of Medical Sciences, Iran; 5755 Yale University, USA; Connecticut Council on Problem Gambling, USA; Connecticut Mental Health Center, USA; Wu Tsai Institute, Yale University, USA; Jönköping University, Sweden; Qazvin University of Medical Sciences, Iran

**Keywords:** Moral distress, nurses, COVID-19 pandemic, systematic review, meta-analysis

## Abstract

**Background:**

Moral distress is a common challenge among professional nurses when caring for their patients, especially when they need to make rapid decisions. Therefore, leaving moral distress unconsidered may jeopardize patient quality of care, safety, and satisfaction.

**Aim:**

To estimate moral distress among nurses.

**Methods:**

This systematic review and meta-analysis conducted systematic search in Scopus, PubMed, ProQuest, ISI Web of Knowledge, and PsycInfo up to end of February 2022. Methodological quality of included studies was assessed using the Newcastle Ottawa checklist. Data from included studies were pooled by meta-analysis with random effect model in STATA software version 14. The selected key measure was mean score of moral distress total score with its’ 95% Confidence Interval was reported. Subgroup analyses and meta-regressions were conducted to identify possible sources of heterogeneity and potentially influencing variables on moral distress. Funnel plots and Begg’s Tests were used to assess publication bias. The Jackknife method was used for sensitivity analysis.

**Ethical consideration:**

The protocol of this project was registered in the PROSPERO database under decree code of CRD42021267773.

**Results:**

Eighty-six manuscripts with 19,537 participants from 21 countries were included. The pooled estimated mean score of moral distress was 2.55 on a 0–10 scale [95% Confidence Interval: 2.27–2.84, I^2^: 98.4%, Tau^2^:0.94]. Publication bias and small study effect was ruled out. Moral distress significantly decreased in the COVID-19 pandemic versus before. Nurses working in developing countries experienced higher level of moral distress compared to their counterparts in developed countries. Nurses' workplace (e.g., hospital ward) was not linked to severity of moral disturbance.

**Conclusion:**

The results of the study showed a low level of pooled estimated score for moral distress. Although the score of moral distress was not high, nurses working in developing countries reported higher levels of moral distress than those working in developed countries. Therefore, it is necessary that future studies focus on creating a supportive environment in hospitals and medical centers for nurses to reduce moral distress and improve healthcare.

## Introduction

Moral distress impacts health care professionals, including nurses, globally.^1^ Moral distress is frequently encountered by professional nurses when caring for patients, especially when they need to make rapid decisions as patient advocates.^2^ Moral distress may co-occur with frustration, anger, and painful emotions, and if left unidentified and unaddressed, may jeopardize patient care, safety, and satisfaction.^3^ Nurses may inadvertently reduce patient support by not fully attending to patient’s suffering and avoiding certain patient requests or needs, thereby undermining health outcomes.^4^ Moral distress has been associated with increased stress, workplace fatigue, impaired inter-professional relationships, job burnout, and reduced nurse satisfaction, and these may ultimately lead to leaving the workplace or leaving the nursing profession and reducing available nursing staff.^2,5^ Moral distress may also reduce nurses’ confidence and abilities to learn and lead to pessimism about, and reduced interests in, nursing.^6^ Accordingly, efforts to reduce moral distress in nurses may lead to improved quality of care.^7^

Complicating approaches to addressing moral distress among nurses, the causes, frequency, and severity of moral distress may vary according to work locations, services provided, and care settings.^8^ Additional factors linked to moral distress in nursing may include feeling the need to provide unnecessary care, having limited physical resources, overwork, observation of patient suffering,^6,9^ beliefs regarding provision of sub-standard care and treatment due to lack of specialist staff and working with poorly qualified people,^10^ inadequate knowledge, fear of talking,^11^ improper inter-professional communication,^12^ caring for critically ill patients, high mortality rates, unfavorable expectations of patients’ families and an inordinate sense of responsibility for patients’ lives and deaths, receipt of inadequate support,^13^ and professional attitudes and psychological characteristics.^14^

Nurses across hospital wards, such as internal medicine, surgery, psychiatry,^9^ oncology,^9,15^ emergency,^16–18^ and specialty wards,^7,19^ may experience moral distress. It has been proposed that nurses in different wards may experience different levels of incidence and severity of moral distress, but evidence are not consistent regarding which kinds of nurses’ experience are more or less linked to moral distress.^20–22^

Several previous reviews have been published on nurses’ moral distress with different designs including systematic reviews,^23–29^ integrative and rapid scoping reviews.^3,30,31^ However, there are limitations regarding these reviews. Of the available reviews, only three summarized the findings using meta-analyses.^23–25^ Also, participants in these studies were limited to one group of nurses including undergraduate nursing students,^26^ oncology,^23^ intensive care unit (ICU),^25^ neonatal and pediatric ICU,^28^ and Iranian nurses.^24^ Therefore, none of previous systematic reviews gathered and compared evidence regarding moral distress across nursing wards. Some previous studies have additional limitations regarding lack of methodological quality assessment and comprehensive literature review.^3,26,27^ Based on the limitations of previous studies, a comprehensive search strategy through main academic databases and gray literature was designed to gather evidence with no limitations regarding nurses’ characteristics including work locations. The other novel aspect of the current systematic review involves the possibility to consider moral distress among nurses before and during the COVID-19 pandemic. Additionally, given that work expectations and conditions may vary across countries with differing levels of development, this was considered in the present study. With the consideration of the literature gaps mentioned above, the current systematic review aimed to estimate moral distress among nurses with subgroup analysis considering characteristics including work location, COVID-19 pandemic timing and development status of the local jurisdiction.

## Methods

### Design and registration

The present study was a systematic review and meta-analysis conducted between October 2021 and February 2022. The protocol of this project was registered in the PROSPERO database affiliated with the International prospective registry of systematic reviews under decree code of CRD42021267773.^32^

### Search strategy

Five academic databases including Scopus, PubMed, ProQuest, ISI Web of Knowledge, and PsycInfo were searched systematically from inception to end of February 2022. To construct the systematic search question and search query, the PECO-S framework was used. Based on PECO, queries were comprised of four aspects: Population (P), Exposure (E), Comparison (C), Outcome (O), and Study design (S).^33^ PECO-S framework in current systematic review was explained as: Nurses for population; working in clinical conditions including hospitals, elderly care setting, health care systems for exposure; comparison was not defined based on the main objective of current systematic review; moral distress mean score was set as outcome; and observational studies including cross sectional or baseline of longitudinal studies were selected study design. Two main components of P (nurse) and O (moral distress) was selected as main search terms. The search terms were extracted from PubMed Medical Subject Heading terms. The main search terms were moral distress and nurses. The search query was developed using the Boolean operators of AND/OR/NOT. The core search syntax was (Nurse OR (Personnel AND Nursing) OR “Nursing Personnel” OR “Registered Nurses” OR (Nurse AND Registered) OR (Nurses AND Registered) OR “Registered Nurse” OR nurse*) AND (“moral distress” OR (moral AND distress) OR “moral stress” OR “moral responsibility” OR “moral dilemma” OR conscience OR “ethical confrontation”). Then search syntax was customized based on the advanced search attributes of each database. Additionally, reference lists of included studies, Open Grey and NYAM were searched as gray literature to increase the comprehensiveness of search.

### Eligibility criteria

Inclusion criteria were considered as below

#### Type of participants

Nurses working in any position and or any clinical setting should be assessed as target population. If nurses were assessed as subgroup of studies, that was included when the findings related to nurses were reported separately.

#### Type of outcomes measure

Moral distress mean scores were considered as the main outcome of current systematic review. So, moral distress should be assessed by valid and reliable scales to be included. Also data on moral distress should be reported as mean and standard deviation (SD).

#### Type of studies

All English, peer-reviewed papers with observational studies including Cross sectional studies or baseline of longitudinal studies published up to February 2022 were included.

### Outcomes

#### Primary outcome

Estimation of moral distress among nurses.

#### Secondary outcomes

1. Comparison of moral distress before and after the COVID-19 pandemic;
2. Influencing variables (e.g., age and working ward) in estimation of moral distress among nurses;
3. Assessment of heterogeneity and possible sources

### Study screening & selection

First, title and abstract of all retrieved papers were screened based on the inclusion criteria. The full texts of potentially relevant studies were further reviewed based on the aforementioned criteria. In this process, relevant studies were selected.

### Quality assessment

The methodological quality (or risk of bias) of included studies was assessed using the Newcastle Ottawa checklist that was developed for appraisal methodological quality of observational studies. Selection, comparability, and outcome were assessed with 7 items. The maximum acquirable score is 9 and scores less than 5 points were classified as being low methodological quality (or having a high risk of bias).^34^ Methodological quality was not considered as an eligibility criterion, but rather its’ impact on pooled effect sizes was examined in subgroup analyses.

### Data extraction

A pre-designed excel sheet form was prepared to extract data including first author’s name, collection date, study design, country, number of participants, percent of female participants, mean age, scale used to assess moral distress, and numerical results regarding the means and standard deviations of moral distress scores. In studies in which nurses were a subgroup of participants, numerical findings related to nurses were extracted.

Three steps of study selection, quality assessment, and data extraction were done independently by two reviewers. In the process, disagreements were resolved through discussion involving the two reviewers.

### Data synthesis

Data from included studies were pooled using quantitative approaches and STATA software version 14. Meta-analyses using random effect models were conducted to include within- and between-study variances.^35^ Statistical heterogeneity was assessed using the Q Cochrane test. The I^2^ index was used to estimate the degree of heterogeneity.^36^ It was interpreted as mild (I^2^ < 25%), moderate (25 < I^2^ < 50%, severe (50 < I^2^ < 75%), and highly severe (I^2^ > 75%).^36^

The selected key measure was mean score of moral distress total score. It was analyzed using Metan module of Stata pooling mean and SDs of included studies. The pooled estimate of this key measure with 95% confidence interval was reported. In the included studies, different versions of the Moral Distress scale with different number of questions and different ranges of acquirable scores were used. But in all studies, higher scores present more moral distress. To have comparable and analyzable scores for the purpose of meta-analysis, the scores obtained from each questionnaire were converted to a scale of 0–10. For this purpose, the average score obtained in the study was multiplied by 10 and then divided by the highest score obtained in that scale. For example, when a mean score of 35 was reported in the range of 0–336, then it was corrected as (35 * 10)/336 = 1.04. Then, 1.04 was used as the corrected mean in a scale ranging from 0–10 and entered in the meta-analysis.

Subgroup analysis (analyzed using Metan module based on subgroups) and meta-regression (analyzed using Metareg module) was done to identify possible sources of heterogeneity and influencing variables on moral distress. Funnel plots (analyzed using Metafunnel module) and Begg’s Tests (analyzed using Metabias module) were used to assess publication bias.^37^ Studies with smaller sample sizes and/or negative or less significant results are often more likely to be less successful to be published.^38^ This may lead to publication bias in a meta-analysis (presented as asymmetric funnel plot and significant Begg’s test).^39^ Presence of publication bias can mislead the conclusions. So, identified publication bias should be corrected using the available methods.^40^ Fill and trim method is one of the best methods to correct publication bias; in which probable related unpublished papers are retrieved using various statistical methods.^41^ In the present study, probable publication bias was corrected using fill and trim method.

The Jackknife method was used for sensitivity analysis (analyzed using Metaninf module).^42^ It is also called “leave one out” method. First, the pooled effect size is estimated from the whole sample. Then, in an iterative process, the pooled effect size is computed when each study is, in turn, dropped from the sample.^43^

## Results

### Study screening & selection process

**Figure 1.**
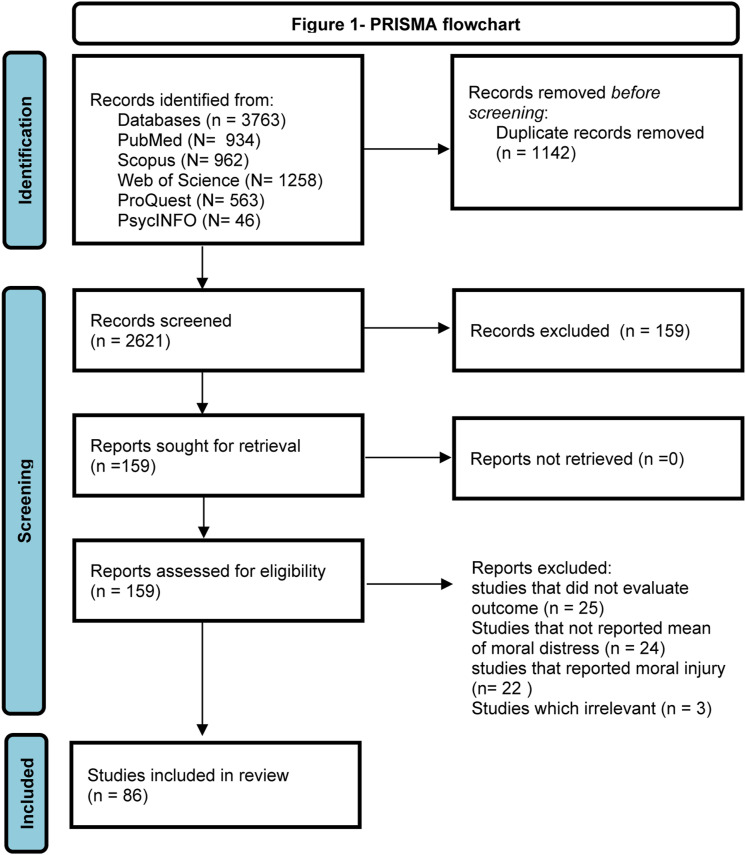
PRISMA flowchart.

The initial search retrieved 3763 studies: PubMed (*N* = 934); Scopus (*N* = 962); Web of Science (*N* = 1258); ProQuest (*N* =563), PsycINFO (*N* = 46). After removing duplicated papers, 2621 papers were screened based on title and abstract and in next stage 159 full text were assessed. Finally, 86 studies met the eligibility criteria and were pooled in the meta-analysis. The search and selection process based on the PRISMA flowchart is provided in Figure 1.

### Study description

86 papers with 19,537 participants from 21 countries (Australia, Brazil, Canada, China, Finland, Germany, Greece, Iran, Israel, Italy, Japan, Netherlands, Norway, Romania, Saudi Arabia, South Africa, Sweden, Thailand, Turkey, UK, and USA) were included. Fifteen papers gathered data during the early part of the COVID-19 pandemic. The smallest sample size was 21 and the largest was 1226. The individual countries with the highest number of eligible studies were Iran (*N* = 27) and USA (*N* = 22). Almost 81% of participants were female. The mean participant age and working experience were 36.28 and 11.52 years, respectively. Most studies were conducted in developed countries (*N* = 49) with a cross-sectional design (*N* = 81). Nine studies were conducted after the onset of the COVID-19 pandemic. Table 1 provides summary characteristics of included studies.

**Table 1.** Summary of characteristics of included studies.

Author	Year	COVID-19 pandemic	Country	Developing status	Design	Working ward	Sample size	Female %	Measure	NOS score
Ventovaara^55^	2021	No	Finland	Developed	Cross sectional	Pediatric oncology	93	95	Moral distress scale–revised	6
Karakachian^56^	2021	No	USA	Developed	Cross sectional	Pediatric	146	91.8	Moral distress scale neonatal–pediatrics	8
Janatolmakan^6^	2021	No	Iran	Developing	Cross sectional	No specific ward	86	40	Moral distress scale–revised	6
Babamohamadi^8^	2021	No	Iran	Developing	Cross sectional	Emergency	203	46.3	Moral distress scale–revised	8
Lake^57^	2021	Yes	USA	Developed	Cross sectional	No specific ward	306	NR	COVID-19 moral distress scale	5
Malliarou^58^	2021	Yes	Greece	Developed	Cross sectional	ICU	257	82.8	Moral distress for healthcare professionals	6
Donkers^59^	2021	Yes	Netherlands	Developed	Cross sectional	ICU	345	77.2	Moral distress for healthcare professionals	5
Prompahakul^60^	2021	Yes	Thailand	Developing	Mixed method	No specific ward	462	97	Moral distress for healthcare professionals	5
Petrisor^61^	2021	No	Romania	Developing	Cross sectional	ICU	79	89.87	Moral distress for healthcare professionals	5
Bleicher^62^	2021	No	USA	Developed	Mixed method	ICU	21	90.5	Moral distress for healthcare professionals	4
Guttmann^63^	2021	No	USA	Developed	Prospective	ICU	178	97	Moral distress thermometer	6
Miljeteig^64^	2021	Yes	Norway	Developed	Cross sectional	No specific ward	525	NR	Moral distress thermometer	6
Tajalli^65^	2021	Yes	Iran	Developing	Cross sectional	NICU	209	NR	Moral distress scale–revised	7
Isık^66^	2021	No	Turkey	Developing	Cross sectional	ICU	128	NR	Moral distress scale–revised	6
Goyaghaj^67^	2021	No	Iran	Developing	Cross sectional	Patients with spinal cord injury	160	61.9	Moral distress scale–revised	6
Fujii^68^	2021	No	Japan	Developed	Cross sectional	No specific ward	242	88	Moral distress for healthcare professionals	6
Nemati^69^	2021	Yes	Iran	Developing	Cross sectional	No specific ward	296	76.07	Moral distress scale	7
Khorashadizadeh^70^	2021	No	Iran	Developing	Cross sectional	No specific ward	454	80.8	Moral distress scale	5
Meese^71^	2021	Yes	USA	Developed	Cross sectional	No specific ward	136	78.26	Single-item moral distress frequency	7
Kok^72^	2020	Yes	Netherlands	Developed	Cross sectional	ICU	194	80.9	Moral distress scale–revised	4
Safarpour^73^	2020	No	Iran	Developing	Cross sectional	No specific ward	217	90.78	Moral distress scale–revised	6
Clark^2^	2020	No	USA	Developed	Cross sectional	Emergency	175	87.4	Moral distress for healthcare professionals	6
Barr^74^	2020	No	Australia	Developed	Cross sectional	NICU	136	99	Moral distress scale neonatal–pediatrics	6
Rezaei Fard^75^	2020	No	Iran	Developing	Cross sectional	No specific ward	150	56	Moral distress scale	7
Marturano^21^	2020	No	USA	Developed	Cross sectional	Oncology	93	93	Moral distress scale–revised	6
Sedaghati^76^	2020	No	Iran	Developing	Cross sectional	Nursing home	227	78	Moral distress scale–revised	7
Sandeberg^77^	2020	No	Sweden	Developed	Cross sectional	No specific ward	223	NR	Moral distress scale–revised	5
Emmamally^78^	2020	No	South Africa	Developing	Cross sectional	CCU	74	NR	Moral distress scale–revised	5
Ramos^79^	2019	No	Brazil	Developing	Cross sectional	No specific ward	1226	NR	Moral distress scale–revised	5
Jones^80^	2019	No	UK	Developed	Cross sectional	Pediatric ICU	1194	95	Moral distress scale–revised	6
Yeganeh^81^	2019	No	Iran	Developing	Cross sectional	ICU	180	93.89	Moral distress scale	5
Sannino^82^	2019	No	Italy	Developed	Cross sectional	Pediatric ICU	136	89	Moral distress scale neonatal–pediatrics	4
Wachholz^83^	2019	No	Brazil	Developing	Cross sectional	No specific ward	141	NR	Moral distress scale–revised	7
Pergert^84^	2019	No	Sweden	Developed	Cross sectional	No specific ward	223	NR	Moral distress scale–revised	7
Bayat^85^	2019	No	Iran	Developing	Cross sectional	No specific ward	300	73.33	Moral distress scale–revised	6
Fruet^86^	2019	No	Brazil	Developing	Cross sectional	Oncology	46	NR	Moral distress scale	6
Zabetian^87^	2019	No	Iran	Developing	Cross sectional	No specific ward	145	90	Moral distress scale	5
Harorani^88^	2019	No	Iran	Developing	Cross sectional	CCU	300	70	Moral distress scale	5
Colville^89^	2019	No	UK	Developed	Cross sectional	ICU	145	77	Moral distress scale–revised	6
Abdolmaleki^90^	2018	No	Iran	Developing	Cross sectional	Emergency	173	NR	Moral distress scale–revised	8
Mehlis^15^	2018	No	Germany	Developed	Cross sectional	Oncology	50	60	Moral distress thermometer	5
Altaker^91^	2018	No	USA	Developed	Cross sectional	ICU	238	90	Moral distress scale–revised	6
Asayesh^92^	2018	No	Iran	Developing	Cross sectional	ICU	117	66.7	Moral distress scale	7
Robaee^93^	2018	No	Iran	Developing	Cross sectional	No specific ward	120	90	Nurses’ MDS by Atashzadeh-Shoorideh	6
Alborzi^94^	2018	No	Iran	Developing	Cross sectional	ICU	100	79	Moral distress scale	6
Lamiani^95^	2018	No	Italy	Developed	Cross sectional	ICU	77	53	Moral distress scale–revised	5
Ajoudani^96^	2018	No	Iran	Developing	Cross sectional	No specific ward	278	85.82	Moral distress scale–revised	8
Neumann^97^	2018	No	USA	Developed	Cross sectional	Transplantation	763	96	Moral distress scale–revised	7
Noghanchi Saleh^98^	2018	No	Iran	Developing	Cross sectional	NICU	172	NR	Moral distress scale	5
Sirilla^99^	2017	No	USA	Developed	Cross sectional	No specific ward	316	NR	Moral distress scale–revised	6
Christodoulou-Fella^100^	2017	No	Europe and the USA	Developed	Cross sectional	Psychiatric	206	56.3	Moral distress scale for mental health services	8
Haghighinezhad^13^	2017	No	Iran	Developing	Cross sectional	ICU	284	33.05	ICU nurses’ moral distress scale	6
Xiaoyan^101^	2016	No	China	Developed	Cross sectional	No specific ward	465	92.25	Moral distress scale–revised	8
Lusignani^102^	2016	No	Italy	Developed	Cross sectional	No specific ward	283	80.21	Moral distress scale–revised	6
Dyo^9^	2016	No	USA	Developed	Cross sectional	No specific ward	279	92.83	Moral distress scale	6
Ameri^103^	2016	No	Iran	Developing	Cross sectional	Oncology	148	88.51	Moral distress scale–revised	5
Ando^104^	2016	No	Japan	Developed	Cross sectional	Psychiatric	130	78.5	Moral distress scale for psychiatric nurses	5
Soleimani^105^	2016	No	Iran	Developing	Cross sectional	No specific ward	193	81.3	Moral distress scale–revised	8
Zavotsky^106^	2016	No	USA	Developed	Cross sectional	Emergency	198	85.4	Moral distress scale–revised	7
Darabzadeh^107^	2016	No	Iran	Developing	Cross sectional	Surgical	123	80.5	Moral distress scale	5
Sauerland^108^	2015	No	USA	Developed	Cross sectional	Pediatric ICU	53	88.7	Moral distress scale neonatal–pediatrics	5
Karagozoglu^109^	2015	No	Turkey	Developing	Cross sectional	ICU	200	73.5	Moral distress scale–revised	5
Trotochaud^110^	2015	No	USA	Developed	Cross sectional	Pediatric	577	NR	Moral distress scale–revised	5
Borhani^111^	2015	No	Iran	Developing	Cross sectional	No specific ward	300	87	Moral distress scale	5
Browning^112^	2015	No	USA	Developed	Cross sectional	No specific ward	277	NR	Moral distress scale	6
de Boer^113^	2015	No	Netherlands	Developed	Prospective	NICU	87	98.9	Moral distress scale–revised	5
Ganz^114^	2014	No	Israel	Developed	Cross sectional	Nurse middle manager	133	88.72	Ethical dilemmas in nursing	6
Sauerland^115^	2014	No	USA	Developed	Cross sectional	CCU	225	80	Moral distress scale	5
Borhani^116^	2014	No	Iran	Developing	Cross sectional	No specific ward	220	NR	Moral distress scale	5
Hamaideh^117^	2014	No	Saudi Arabia	Developing	Cross sectional	Psychiatric	130	56.9	Moral distress scale for psychiatric nurses	6
Karanikola^118^	2014	No	Italy	Developed	Cross sectional	ICU	566	70.8	Moral distress scale	7
Shoorideh^119^	2014	No	Iran	Developing	Cross sectional	ICU	159	72.3	ICU nurses’ moral distress scale	5
Wilson^120^	2013	No	USA	Developed	Cross sectional	ICU	48	94	Moral distress scale	6
Ganz^121^	2013	No	Israel	Developed	Cross sectional	ICU	291	75.3	Moral distress scale	7
Fernandez-Parsons^122^	2013	No	USA	Developed	Cross sectional	Emergency	51	NR	Moral distress scale–revised	5
Ganz^123^	2012	No	Israel	Developed	Cross sectional	Surgical	119	93.3	Ethical dilemmas in nursing	7
Lazzarin^124^	2012	No	Italy	Developed	Cross sectional	Oncology and hematology	235	95	Moral distress scale neonatal–pediatrics	4
Papathanassoglou^125^	2012	No	European critical care conference delegates	Developed	Cross sectional	ICU	255	NR	Moral distress scale	7
Villers_ Non-CCU nurses^126^	2012	No	USA	Developed	Cross sectional	No specific ward	28	96	Moral distress scale	6
Villers_CCU nurses^126^	2012	No	USA	Developed	Cross sectional	CCU	65	91	Moral distress scale	6
Joolaee^127^	2012	No	Iran	Developing	Cross sectional	No specific ward	210	90	Moral distress scale	5
McAndrew^128^	2011	No	USA	Developed	Cross sectional	ICU	78	NR	Moral distress scale	7
Ohnishi^129^	2010	No	Japan	Developed	Cross sectional	Psychiatric	264	73.1	Moral distress scale for psychiatric nurses	6
Pauly^130^	2009	No	Canada	Developed	Cross sectional	No specific ward	374	94	Moral distress scale–revised	6
Elpern^131^	2005	No	USA	Developed	Cross sectional	ICU	28	77	Moral distress scale	5

ICU: Intensive care unit, NICU: Neonatal intensive care unit, PICU: Pediatric intensive care unit, CCU: Cardiac care unit; NOS score: Methodological quality score based on NOS checklist.

### Methodological quality assessment

**Figure 2.**
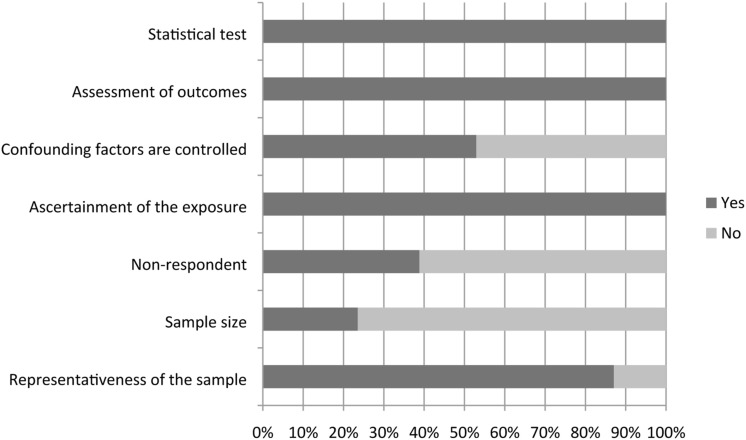
Results of the methodological quality assessment based on the NOS checklist.

Considering Newcastle Ottawa scores (NOS) > 5 as high quality, 65.88% of included studies (56 papers) were categorized as having low risk of bias. Methodological problems were related to: (i) no explanations regarding sample size estimation; (ii) no explanations regarding non-respondents and how non-response was managed; and (iii) controlling for potentially confounding factors. Figure 2 provides results of methodological quality assessments based on NOS checklist items.

### Estimation of pooled moral distress mean score

**Figure 3.**
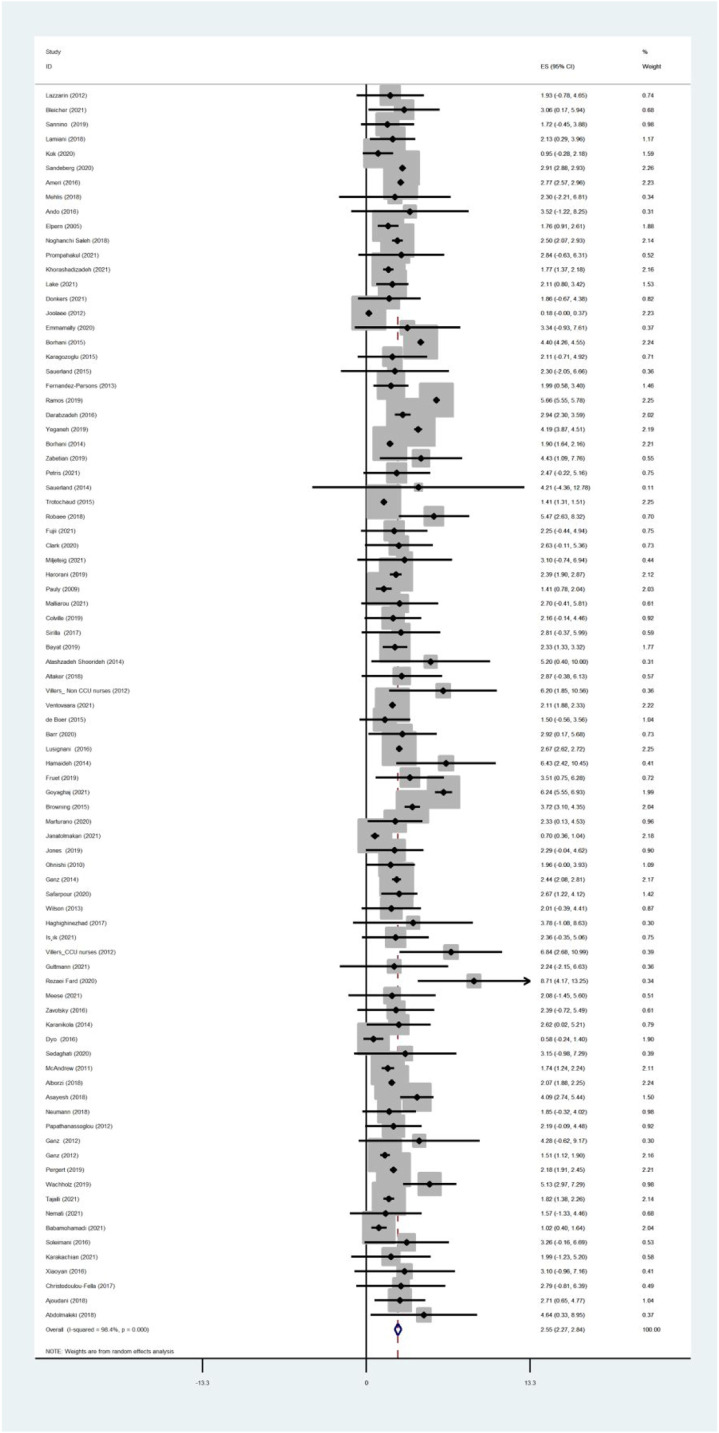
Forest plot of estimated pooled mean scores of moral distress.

**Figure 4.**
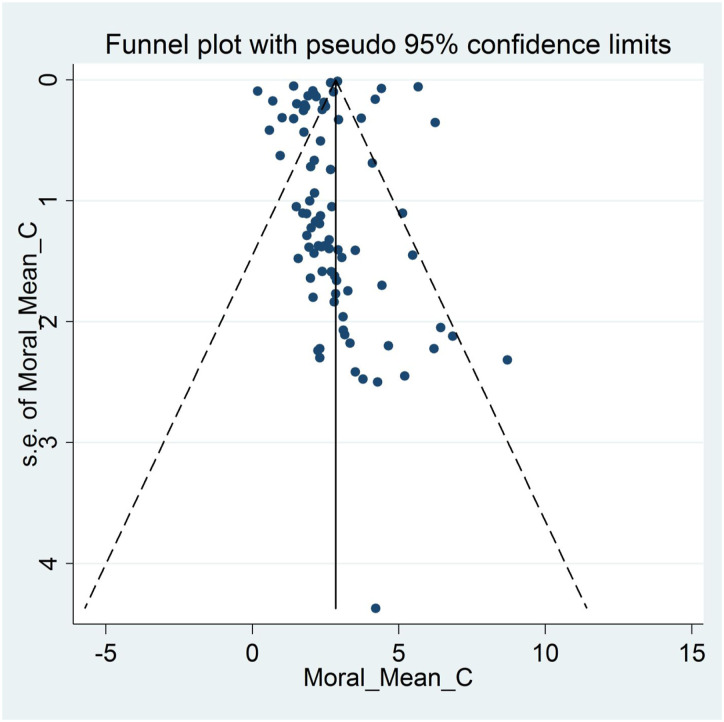
Funnel plot assessing publication bias in estimated pooled mean scores of moral distress.

The pooled estimated mean score of moral distress was 2.55 in a range of 0–10 [95% Confidence Interval: 2.27–2.84, I^2^: 98.4%, Tau^2^:0.94]. Figure 3 provides a forest plot regarding the pooled estimated mean score of moral distress. Begg’s tests (*p* < .001) and funnel plots (Figure 4) consider probabilities of publication bias. Meta trim was used to correct for probable publication bias. But on trim methodology, no studies were imputed and probability of publication bias was considered low. Also, sensitivity analysis suggested that the pooled effect size was not affected by any single study.

### Subgroup/meta-regression results

The results of subgroup analysis (Table 2) and meta-regression (Table 3) showed that mean score of moral distress significantly decreased after the onset of the COVID-19 pandemic (1.80 vs 2.62). Nurses working in developing countries experienced higher levels of moral distress compared to their counterparts in developed countries (3.14 vs 2.14). Nurses in developed countries experienced less moral distress than their counterparts in developing countries by 0.76 point lower on a scale of 0–10, according to meta-regression analysis (*p* = .02). The variables of methodological quality and study design had no significant effect on the mean score of moral distress (*p* > .05). Nurses’ workplace location had no significant relationship with moral distress (*p* = .62). However, the lowest mean scores of moral distress were observed in pediatric and emergency ward nurses (1.41 and 1.60, respectively), and the highest scores were observed in critical care unit and psychiatric ward nurses (3.42 and 3.14, respectively). Also, the workplace ward had the greatest effect on heterogeneity. The lowest heterogeneity was observed in psychiatry and emergency wards (23.4% and 33%). Among the investigated variables, country’s development status and nurses’ workplace explained 5.59% and 3.16% of the variance in moral distress among nurses.

**Table 2.** Results of subgroup analyses.

Variable		No. of studies	ES (95% CI)	I^2^ (%)	Tau^2^
COVID-19 pandemic	Prior	76	2.62 (2.32; 2.92)	98.5	0.4
	During	9	1.80 (1.42; 2.18)	0	0
Developmental status	Developed	49	2.14 (1.88; 2.38)	95.3	0.26
	Developing	36	3.14 (2.39; 3.89)	99.1	4.10
Design	Cross sectional	83	2.57 (2.28; 2.85)	98.4	0.97
	Prospective	2	1.63 (−0.23; 3.50)	0	0
Methodological quality	Low risk of bias	56	2.47 (2.17; 2.77)	87.9	0.05
	High risk of bias	29	2.53 (1.91; 3.14)	99.4	1.98
Working ward	Oncology	6	2.45 (1.91; 2.99)	74.8	0.16
	Pediatrics	3	1.41 (1.31; 1.51)	0	0
	Emergency	4	1.60 (0.62; 2.59)	33.0	0.35
	ICU (including NICU & PICU)	29	2.31 (1.83; 2.79)	84.8	0.82
	CCU	4	3.42 (1.37; 5.48)	35.8	1.76
	Psychiatric	4	3.14 (1.27; 5.01)	23.4	0.90
	No specific wards	35	2.80 (2.38; 3.22)	99.1	0.96
Overall estimated prevalence		85	2.55 (2.27; 2.84)	98.4	0.94

ICU: Intensive care unit, NICU: Neonatal intensive care unit, PICU: Pediatric intensive care unit, CCU: Cardiac care unit.

**Table 3.** Results of meta-regression.

Variable	Number of studies	Coeff	S.E.	*p*	I^2^ res. (%)	Adj. R^2^ (%)	Tau^2^
Country	85	0.01	0.03	0.65	98.37	−0.67	1.30
Mean age	47	0.005	0.03	0.88	97.41	−2.10	1.57
Mean working experience	38	0.01	0.06	0.85	94.24	−3.60	1.39
Female percentage of participants	66	−0.002	0.01	0.91	96.37	- 2.45	1.32
Working ward	85	0.07	0.05	0.18	98.35	3.16	1.83
Methodological quality score	85	−0.06	0.17	0.75	98.28	−0.57	1.31
Development status (developed vs developing)	85	−0.76	0.32	0.02	98.26	5.59	1.22
COVID-19 pandemic (during vs prior)	85	−0.74	0.56	0.19	98.38	0.76	1.28
Measure assessing moral distress	85	0.06	0.07	0.36	98.37	−1.06	1.31

Coeff: Coefficient; S.E: Standard Error; I^2^ res: I^2^ residual; Adj. R^2^: Adjusted R^2^.

## Discussion

Given the importance of moral distress among healthcare professionals (e.g., healthcare professionals may respond sub-optimally to certain patient requests or needs based on moral distress, and this result in poor health outcomes for patients),^1–5^ it is important to understand moral distress among healthcare professionals, especially nurses who often interact more frequently with patients in acute care settings than do other healthcare professional. The present systematic review and meta-analysis used rigorous methods (including a thorough search of five commonly used academic databases, the use of the NOS to evaluate and control for study quality in meta-analysis,^34^ the application of subgroup analyses and meta-analyses to identify potential sources of heterogeneity, and several statistical methods to assess and correct fir possible publication bias^37,44^).

Data from 19,537 participants reported in 86 papers across 21 countries (Australia, Brazil, Canada, China, Finland, Germany, Greece, Iran, Israel, Italy, Japan, Netherlands, Norway, Romania, Saudi Arabia, South Africa, Sweden, Thailand, Turkey, UK, and USA) were assessed, and the mean moral distress was 2.55 on a 0–10 scale. This indicates that in general the nurses did not have high levels of moral distress in their clinical practices. Moreover, such findings were found to be consistent across high-quality versus low-quality studies. The relatively low moral distress suggests that moral distress may not interfere too frequently with nurses provision of quality care.^7^ However, subgroup analyses in the present systematic review and meta-analysis revealed that nurses working in developing countries had higher levels of moral distress than those working in developed countries. Moreover, nurses working in a critical care unit or a psychiatric ward appeared to have higher levels of moral distress than those working in other wards (especially those working in pediatric ward), although this finding was not statistically significant. Thus, identifying and addressing moral distress among nurses may be particularly relevant for those working in developing countries.

Nurses in the developing countries may encounter unique experiences including with respect to poor availabilities of health care equipment, training programs, and standardized care procedures when compared to those in the developed countries.^45–48^ For example, prior evidence shows that the healthcare infrastructure in developing countries may not be capable to of optimally supporting health information systems, mHealth, and artificial intelligence technologies.^46–48^ Therefore, as compared with nurses in developed countries, nurses working in a developing country may have more difficulties in providing immediate and state-of-the-art treatments to patients. Moral distress may thus occur when nurses in developing country experience limitations in providing high-quality care although this notion is currently speculative and requires direct examination. Moreover, healthcare budgets in developing countries are frequently low and focus on communicable diseases.^49^ Therefore, nurses in developing countries may encounter shortages of resources in healthcare settings. Consequently, nurses in developing countries may be likely to suffer from moral distress than those in developed countries, and these possibilities warrant further examination.

Nurses working in a critical care unit or a psychiatric ward were found to have numerically high levels of moral distress. This may reflect difficulties and complexities of caring for patients with critical needs or psychiatric conditions.^50,51^ Caring for patients with critical needs is often associated with burdens of uncertainty and difficulties in treatment-related decision-making.^52^ Speculatively, such difficulties in decision-making may increase moral distress among nurses. For nurses providing psychiatric care, they often experience stigma (e.g., affiliated stigma leading to self-stigma),^53,54^ and subsequently, nurses providing psychiatric care may be more likely to escape from feelings of stigma via providing less optimal care to patients, further generating moral distress. These currently speculative possibilities warrant direct examination.

Finally, the observation that moral distress among nurses have not increased during the onset of the COVID-19 pandemic is heartening and suggests that nurses may have specific resiliency to mitigate against moral distress during the COVID-19-related circumstances. Identification of resiliency factors is important as they may help guide interventions and prevent moral distress and related factors like burnout.

### Limitations

There are limitations in the present systematic review and meta-analysis. First, the population was restricted to nurses. Given that nurses and other healthcare professionals may encounter different moral distress in clinical practice, the findings of the present study may not generalize to other healthcare professionals. However, the focus on nurses is important as they often interact frequently with patients in healthcare settings. Also, it should be considered that the general term of nurse (who passed academic courses and graduated as nurse) and its’ MeSH terms were used to develop search syntax. In many countries, different levels of nurses exists—Registered nurses, licensed practical nurses, and auxiliaries and similar. Most of these terms used for nurses and nursing personnel are retrievable by the comprehensive search syntax developed for current study. But it should be noted that if terms other that nurse were used, those studies might not be retrieved. Second, only studies published in English were included in the present systematic review and meta-analysis. Therefore, some data published in other languages may have been omitted. Third, most included papers utilized a cross sectional study design, therefore, limiting insight into potential causal factors relating to moral distress. Future studies with longitudinal designs are warranted.

### Clinical implication

The findings of the present systematic review and meta-analysis suggest the following implications for nursing management. First, moral distress among nurses was found to be higher in developing countries than in developed countries. Therefore, nurse managers, administrators, and other stakeholders should attend to moral distress among nurses, particularly those working in developing countries. Regular workshops helping nurses to overcome moral distress may be important to target moral distress among nurses. Second, nurses working in some specific wards (e.g., critical care unit and psychiatric wards) may experience high levels of moral distress. Therefore, evaluating and addressing moral distress in these settings may be particularly important. In all cases, identifying risk and resilience factors related to moral distress among nurses appears important. Such information may help with developing and targeting appropriate interventions to reduce moral distress among nurses.

## Conclusion

In conclusion, the present systematic review and meta-analysis showed a low the pooled estimated score of moral distress. Although the score of moral distress was not high, nurses working in developing countries encountered higher levels of moral distress than those working in developed countries. Nurses in developing countries face many challenges that can affect their moral distress. Therefore, it is necessary that future studies focus on creating a supportive environment in hospitals and medical centers for nurses to reduce moral distress and improve healthcare.
